# Rapid detection of anti-Vaccinia virus neutralizing antibodies

**DOI:** 10.1186/1743-422X-8-139

**Published:** 2011-03-25

**Authors:** Marit Kramski, Anna Drozd, Gregor F Lichtfuss, Piotr W Dabrowski, Heinz Ellerbrok

**Affiliations:** 1 Robert Koch-Institute, Centre for Biological Safety, Nordufer 20, 13353 Berlin, Germany; 2 Department of Microbiology and Immunology, University of Melbourne, Royal Parade, Parkville, 3206 VIC, Australia; 3 Burnet Institute, 85 Commercial Road, Melbourne, Australia

## Abstract

Increasing infections with Monkeypox and Cowpox viruses pose a continuous and growing threat to human health. The standard method for detecting poxvirus neutralizing antibodies is the plaque-reduction neutralization test that is specific but also time-consuming and laborious. Therefore, a rapid and reliable method was developed to determine neutralizing antibody titers within twelve hours. The new assay measures viral mRNA transcription as a marker for actively replicating virus after incomplete neutralization using real-time PCR.

## Background

The increasing number of humans infected with zoonotic orthopox viruses (OPV) such as Cowpox and Monkeypox virus poses a continuous and increasing threat to human health [1]. Thus, efforts to develop new vaccines against OPV infections remain important. Natural immune response and efficacy of vaccines are characterized through their capacity to induce neutralizing antibodies. The standard diagnostic method to determine OPV neutralizing antibodies in plasma or serum samples is the plaque-reduction neutralization test (PRNT). It quantifies neutralizing antibodies by measuring the reduction of virus-induced plaques where one infectious virus particle is directly related to one virus-formed plaque. PRNT is the gold standard because it is specific, direct and reproducible [2]. However the PRNT suffers from long turn-around times (several days), is laborious and uses an operator-error prone manual readout based on calculating the neutralization titer from the number of plaques counted. Due to long incubation times of the infected cell cultures necessary to allow plaque formation, anti-co-agglutinants like EDTA and plasma components can interfere with the cell monolayer and affect plaque formation, especially in low plasma dilutions. While pre-dilution of plasma might reduce these effects, it also reduces sensitivity of the PRNT and low titers of neutralizing antibodies might be missed.

Recently, four alternative methods were described to determine neutralizing anti-Vacinia virus (VACV) antibodies using either a beta-galactosidase expressing VACV Western Reserve strain (WR) [3] or recombinant GFP expressing VACV strains [4,5]. Eyal et al. [6] measured remaining infectivity by enzyme immunoassay using VACV strains WR and Lister Elstree (LE). These assays are designed for large-scale screening but still are time consuming. Additionally, three of them require the use of specific recombinant VACV strains.

The assay presented here uses VACV LE and human VACV immunoglobulin (HIVIG) as a model system and quantifies neutralizing anti-VACV antibodies by combining the classic PRNT with a OPV-specific real-time PCR (designated NT-PCR) allowing quantification of replicating virus within a few hours after infection of the host cell.

## Results and discussion

### Validation of real-time PCR assays

To quantify actively replicating virus, three OPV-specific reverse transcriptase real-time PCR assays were established. The OPV_12/13 _assay targets the gene for the VACV LE DNA-binding phosphoprotein involved in DNA replication and nucleotide metabolism. The other two OPV-specific real-time PCR assays, D8L and Rpo18 [7], are specific for the D8L membrane protein coding region of IMV particles and the 18-kDa RNA polymerase subunit gene, respectively. All three real-time PCR assays were OPV-specific, showed no cross-reactivity to cellular genes (data not shown) and therefore were used as a measure for replicating virus within cells. To standardize virus mRNA copies to an equal number of cells a cellular gene-specific c-myc real-time PCR assay was used. All assays have a linear detection range from 10^6 ^to 10 copies per reaction with an overall R^2 ^of ≥ 0.98 and PCR efficiencies ≥ 95% (table 1), which are common features for many other real-time PCR assays used in virology and microbiology [8-10]. Results of intra- and inter-assay variability for plasmids standards were less than 1 C_T _(see details for OPV_12/13 _and c-myc assays in table 1) demonstrating a high degree of intra- and inter-assay precision.

**Table 1:** Variability and efficiency of OPV_12/13 _and c-myc real-time PCR assays.

PCR Assay	copy no.	Variability* C_**T**_		**R**^2 ^**	PCR Efficiency (%) **
				
		intra-assay	inter-assay		
**OPV _12/13_**	10^6^	17.58 ± 0.23	17.42 ± 0.20	0.99	96.00
	10^5^	20.33 ± 0.16	20.55 ± 0.20		
	10^4^	24.13 ± 0.15	24.34 ± 0.21		
	10^3^	27.01 ± 0.08	27.42 ± 0.41		
	10^2^	31.22 ± 0.08	31.24 ± 0.38		
	10^1^	34.42 ± 0.05	34.96 ± 0.47		
**c-myc**	10^6^	19.63 ± 0.04	19.74 ± 0.46	0.99	97.62
	10^5^	23.23 ± 0.28	23.09 ± 0.31		
	10^4^	26.40 ± 0.05	26.61 ± 0.29		
	10^3^	29.53 ± 0.02	29.65 ± 0.55		
	10^2^	34.73 ± 0.49	33.76 ± 0.97		
	10^1^	36.74 ± 0.36	36.39 ± 0.58		

* for the determination of the intra-assay variability, plasmid standards (10^6^-10^1 ^plasmid copies) were measured in triplicate, and for the inter-assay variability, measurements were repeated on 3 consecutive days; ** calculation using the slope of the calibration curves for plasmid standards (10^1^-10^6 ^plasmid copies); The linear detection range is 10^6^-10^1 ^copies per run. C_T _threshold cycle

### Evaluation of HVIG neutralizing antibody titers with standard PRNT

Neutralizing antibody titers of HVIG (VIG and Omrigam) were first determined with the standard PRNT. The PRNT titer is defined as the antibody dilution resulting in 50% plaque reduction. HVIG preparations were tested using 4.4 × 10^1 ^pfu/well VACV LE and 1 h, 2 h or 3 h of incubation for virus neutralization. For both, VIG and Omrigam, the mean neutralizing PRNT titer from three independent measurements was 1:320± one dilution step. VIG neutralizing titers varied depending on neutralization time: 1:160 (n = 1/3 for 1 h neutralization, n = 1/3 for 2 h neutralization), 1:320 (n = 2/3 for 1 h, 2 h and 3 h neutralization) and 1:640 (n = 1/3 for 3 h neutralization). The PRNT titer for Omrigam was always 1:320 (n = 3 for 1 h, 2 h and 3 h of neutralization). As both HVIG showed the same neutralizing activity in the PRNT, VIG was used for establishment of the NT-PCR assay for reason of availability.

### The NT-PCR assay

The NT-PCR assay principle is based on measuring actively replicating VACV LE by quantifying virus mRNA levels in infected Vero E6 cells. Since cell numbers per well, RNA integrity and quantity can vary all virus copy numbers were normalized against 10^6 ^copies of the cellular reference gene transcript of c-myc. The c-myc gene is expressed constitutively and independently from experimental conditions and sample treatment, different cell types and developmental stages. It is not affected by the infection with different OPVs (A. Nitsche, personal communication). After pre-incubation of VACV LE with VIG fewer cells became infected compared to VACV LE alone resulting in a decreased virus mRNA copy number due to neutralization of infectious virus. For VACV LE incubated with negative human serum virus mRNA copy numbers were set to 100%. Based on the mRNA copy numbers determined for each antibody dilution the percentage of replicating virus was calculated in comparison to the 100% expression in virus control sample.

For the NT-PCR assay virus concentrations of 4.4 × 10^1^, 2.2 × 10^2 ^and 4.4 × 10^2 ^pfu/well were tested using experimental conditions similar to the standard PRNT with 1 h neutralization time and 24 h for infection and replication in order to compare both assays. Using 4.4 × 10^1 ^and 2.2 × 10^2 ^pfu/well of VACV LE, levels of viral mRNA were below detection threshold of the OPV_12/13 _real-time PCR assay (< 10^1 ^copies) for antibody dilutions 1:40-1:80. For antibody dilutions ≥ 1:160 copy numbers varied between 10^1 ^and 10^3 ^copies. No correlation between virus replication and antibody dilution was observed. In contrast, when using a virus dose of 4.4 × 10^2 ^pfu/well, ≥ 10^1 ^OPV_12/13 _copies were detected for all antibody dilutions tested. A significant increase of virus replication could be detected for an antibody dilution of ≥ 1:640. Therefore, an infectious dose of 4.4 × 10^2 ^pfu was selected for further experiments.

To reduce handling time, neutralization efficacy was evaluated after 1 h, 2 h and 3 h of co-incubation of VACV LE with the VIG antibodies. For 1 h and 3 h of co-incubation no significant correlation was detected due to high variability of virus replication for all antibody dilutions. After 2 h a significant increase of virus replication was observed correlating with increasing antibody dilution. Virus mRNA copy numbers revealed ≤ 3x10^2 ^OPV_12/13 _copies for antibody dilutions < 1:640 whereas copy numbers for dilutions ≥ 1:640 increased at least twofold (data not shown). A visual cut-off was set for a dilution of 1:640 and neutralization time of 2 h was chosen for further validation.

While a complete replication cycle of OPV takes between 9 and 24 h [11,12], early gene expression is detectable by real-time PCR less than 30 min after infection [13]. In initial experiments virus was allowed to replicate for 24 h to ensure one complete replication cycle. However, to reduce assay time, expression of viral genes was evaluated after 5 h and 7 h of virus infection. Additionally to the OPV_12/13 _assay, two other real-time PCR assays (Rpo18, D8L) were tested simultaneously. While all three assays target genes highly conserved between OPV species only OPV_12/13 _and Rpo18 target genes directly involved in virus replication. Although all tested OPV-specific real-time PCR assays have similar characteristics in terms of sensitivity, amplification efficiency and specificity no significant correlation between antibody dilution and virus neutralization could be seen with the D8L real-time PCR assay (data not shown). Thus, the D8L assay was not used for further experiments. In contrast, after 7 h but not after 5 h of virus replication, the Rpo18 and OPV_12/13 _real-time PCR assays were successfully and consistently detecting increased viral mRNA levels for antibody dilutions ≥ 1:1280 and 1:640, respectively, indicating reduced virus neutralization. Using the OPV_12/13 _and Rpo18 assay a significant correlation between antibody dilution and virus replication was detected. The results for OPV_12/13 _and Rpo 18 suggest that it is important to use a real-time PCR that targets a gene directly involved in virus replication. As the OPV_12/13 _assay was originally designed to detect the DNA binding phosphoprotein of VACV the OPV_12/13 _and an infection time of 7 h was selected for further validation of the NT-PCR.

**Figure 1:**
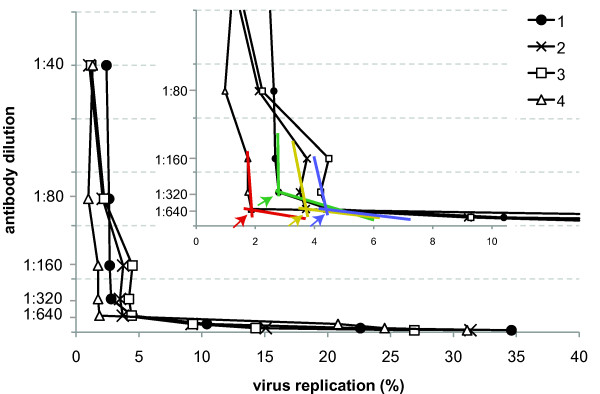
**Graphical illustration of deriving the NT-PCR neutralizing titer in a normal analytical setting**. Instead of performing statistical analysis of multiple runs (as in figure 2) the point at which the slope of the curve shows a sudden decrease to a near-zero value identifies the point of significant increase of virus replication caused by incomplete virus neutralization (if antibody dilution is plotted against percent virus replication) and defines the neutralizing antibody titer. Mathematically, this point can be derived by treating the curve as a composition of two near-linear fragments (colored curves). The intersection (arrows in enlarged insert), which results in the best linear fit (R2) on both modeled linear segments (= highest R2a+R2b) defines the NT-PCR titer. Numbers indicate curves derived from different number of replicates per run: #1 and #2: mean values of two wells; #3: mean value of three wells; #4: mean value of four wells analyzed per dilution step, respectively. Independent of the number of replicates analyzed the resulting neutralization titer was always the same, suggesting that duplicate measurements should suffice for normal clinical or analytical application of the NT-PCR assay.

To ensure a qualitative and repetitive readout, the neutralizing antibody titer (= NT-PCR titer) was calculated based on a plot of the antibody dilution versus percentage of virus replication: the point at which the curve becomes near-horizontally (the slope approaches a near-zero value) defines the NT-PCR titer (Figure 1). This point is characterized by incomplete virus neutralization with significant increase in virus replication as a result of antibody dilution. Mathematically this point can be identified by defining the titration curve as a combination of two linear segments (lower than dilution x (segment a) and higher than dilution x (segment b)) and using linear regression to identify the intersection (dilution x) that leads to the best fits on both segments: The intersection (dilution x), that results in the best linear fit (R2) on both modeled linear segments (= highest R2a+R2b), defines the NT-PCR titer.

**Figure 2:**
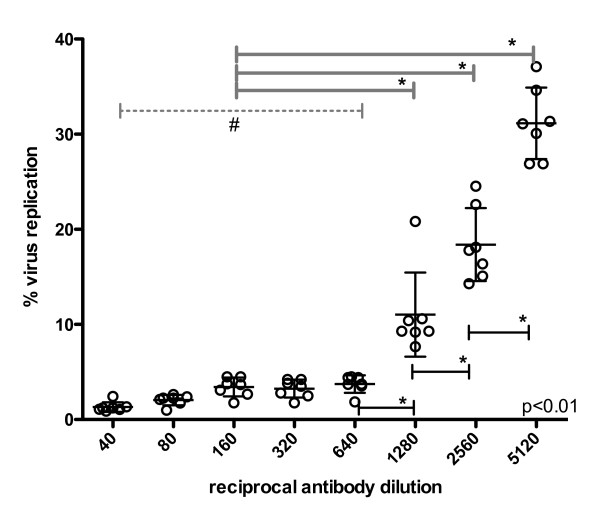
**Determination of neutralizing antibody titer as established using the NT-PCR**. Percent virus replication is plotted against antibody dilution and the neutralizing titer is defined by the last antibody dilution step before virus replication is significantly increasing (≥1:640). Data is shown for seven individual runs using mean values from two, three and four single wells. Bars indicate means and SD, as a normal distribution of the repeated measures was assumed. The p-value indicates significant differences across all compared dilution groups (significance indicated by *): # the grey dotted line indicates the range of dilutions from 1:40 to 1:640 where no significant differences between the individual dilution steps are observed, however all individual columns are significantly different to dilutions ≥ 1:640 (1:1280 or higher).

Intra-assay variation of the NT-PCR was determined based on four runs performed in parallel. To evaluate inter-assay variability the NT-PCR assay was performed on four different days. Calculation of the NT-PCR titer was performed for a total of seven assays (table 2). The NT-PCR is designed as a single well format assay and measures the inhibition of virus replication for a particular antibody dilution in one well. However, to decrease the variability between samples on the same plate the actual NT-PCR titer was calculated using the means of % virus replication of either two (for 5 out of 7 assays), three (for 1 out of 7 assays) or four (for 1 out of 7 assays) single wells which were set up in parallel and treated identically. Comparison of the cut-off values (= NT-PCR titer) resulted in an intra- and inter-assay variability of 1:640 ± one dilution step (table 2, Figure 2) independent of the number of single wells used to calculate the mean value. Statistical analysis indicates that dilutions from 1:40 to 1:640 where not significantly different to each other. However, all individual dilutions between 1:40 and 1:640 are significantly different to dilutions ≥ 1:640. This further shows that the introduction of a quantitative PCR allows the reliable calculation of a neutralizing antibody titer that is not exclusively based on a visual readout as in the case of the PRNT assay which might be subject to operator error.

**Table 2:** Calculation of the neutralizing antibody titer determined with NT-PCR assay (1.) and its intra- and inter-assay variability (2.)

*1. Calculation of the NT-PCR assay*											
		**Σ R**^**2 **^single well*									
	
**antibody dilution**		**assay #**	**1**^**a**^	**2**^**a**^	**3**^**a**^	**4**^**a**^	**5**^**a**^	**6**^**b**^	**7**^**c**^		
	
1:40	0.0250		0.25	0.31	0.27	0.33	0.22	0.32	0.31		
1:80	0.0125		1.34	1.37	1.31	1.37	1.26	1.35	1.42		
1:160	0.0063		1.44	1.36	1.28	1.27	1.30	1.23	0.71		
1:320	0.0031		**1.61**	1.52	1.45	1.49	1.17	1.44	1.11		
**1:640**	**0.0016**		1.20	**1.77**	**1.65**	**1.73**	**1.41**	**1.65**	**1.43**		
1:1280	0.0008		1.23	1.34	1.30	1.54	1.30	1.41	1.06		
1:2560	0.0004		1.24	1.36	1.39	1.38	1.31	1.46	1.26		
1:5120	0.0002		0.25	0.31	0.27	0.33	0.22	0.32	0.31		

***2. Inter-and intra-assay precision***											

		respective NT-PCR titer for each assay									
			
		day 1		day 2		day 3		day 4		inter-assay variability	
			
		assay 1	1:320	assay 3	1:640	assay 4	1:640	assay 5	1:640	1:640± one dilution	
		assay 2	1:640								
		assay 6	1:640								
		assay 7	1:640								
									
intra-assay variability		1:640± one dilution									

*R^2 ^is the R-squared value (R-squared value is the fraction of the variance in the data that is explained by a regression); ΣR^2 ^is the sum of R1^2 ^and R2^2^; **single well sample 1-7**: ^a ^mean value resulting from two wells; ^b ^mean value resulting from three wells; ^c ^mean value resulting from four wells; NT-PCR antibody titer is highlighted in bold and underlined and corresponds to the highest ΣR^2^

To explore the NT-PCR assay's application for clinical use, serum samples of six individuals recently vaccinated with VACV LE were tested using both, PRNT and NT-PCR. As only small serum volumes were available, serial dilution of serum was started at 1:40. For two of the six serum samples the outcome was identical in both tests (table 3). However, the NT-PCR and PRNT titers for three serum samples differed between one and two dilution steps still in the range of normal cell culture assay variability. Patient sample no. 6 was considered to be an outlier, as neutralizing antibody titers determined with both assays could not be confirmed due to lack of sample volume. Neutralizing titers are not identical as both assays have a different readout and definition of the cut-off of the titer. Moreover, the limited number and small sample volumes of sera available prevented further evaluation of the NT-PCR assay as well as statistical comparison.

**Table 3:** Serum samples from VACV LE vaccinated individuals analyzed by PRNT and NT-PCR.

Patient #*	NT-PCR titer	PRNT titer
1	1:80	1:40
2	1:160	1:40
3	1:160	1:160
4	1:320	1:640
5	1:40	1:40
6^§^	1:640	1:40

* lowest dilution step was 1:40, bigger volumes of serum were not available

§ result for this patient is considered as outlier (PRNT was done only once because of lack of serum availability)

Although further evaluation with clinical samples is needed this preliminary evaluation indicates that it will be suitable for routine diagnostic use especially if results are needed rapidly, if plasma components interfere with the cell culture or if neutralizing antibodies to viruses with no or poor cytopathic effect in cell culture (no need for the detection of plaques) need to be analyzed. It should be easily adaptable to other DNA viruses as soon as a suitable real-time PCR assay is available to detect viral mRNA. For DNA viruses the detection of viral mRNA indicates the presence of actively replicating virus in the cell limiting the potential effect of contaminating DNA from the genome of the inoculated virus just sticking to the cell surface. Also, the assay should be adaptable to RNA viruses if active virus replication could be determined and distinguished from mere contamination from viral genomic RNA. Assay conditions need to be adapted for each new virus, however, this is also true for standard PRNT assays.

Taken together, the new and rapid NT-PCR assay can be completed within 12 h while the turn-around time of the standard PRNT is at least 4 days. Final NT-PCR conditions are: 4.4 × 10^2 ^pfu/well VACV LE, 2 h of neutralization time, 7 h of infection and replication time and final quantitative examination of virus mRNA copies in cells by quantitative OPV_12/13 _real-time PCR.

## Conclusions

The NT-PCR assay is a fast, reliable and sensitive tool for the detection of VACV neutralizing antibodies. It will be suitable for routine diagnostic and could pose a valuable alternative to the standard PRNT assay. The low detection limit and the high degree of accuracy for the detection of replicating virus by real-time PCR resulted in high reproducibility and short assay turn-around time. This assay can be performed with all wild-type OPV strains without the need of particular VACV reporter strains. It may also be beneficial for viruses with no or poor cytopathic effect in cell culture.

## Methods

### Cells, viruses and antibodies

VACV LE was propagated in Hep2 cells (ECACC: 86030501). Vero E6 cells (ECACC: 85020205) were used for virus stock titrations, for standard PRNT and NT-PCR. Overlay medium was 1.6% carboxymethylcellulose (CMC). All cells were maintained according to ECACC recommendations.

VACV LE (Bavarian Nordic, Martinsried, Germany) (4.4 × 10^8 ^pfu/mL), human Vaccinia virus immunoglobulin (HVIG) (VIG antibodies, BER/FDA Bethesda, MD, USA and Omrigam 5%, G2H50CN HO4021, Israel, 2003) and sera from six individuals vaccinated with VACV LE were used. All sera were tested for absence of cytotoxicity.

### PRNT

For standard PRNT 8.5 × 10^4 ^cells/well of Vero E6 cells were seeded into 48-well plates and incubated over night. HVIG and sera were twofold serially diluted in cell culture medium from 1:40 to 1:2560 and mixed with an equal volume of VACV LE to 4.4 × 10^1 ^pfu/100 μL. Medium served as negative control. After 1 h 100 μL of serum-virus mixture was added to cells in 100 μL cell culture medium. Plates were centrifuged (15 min, 200 × g) and virus was allowed to adsorb for 1 h at 37°C. Next, cells were overlaid with 400 μL overlay medium and incubated for 4 days. Medium was aspirated and cells were fixed with 4% formaldehyde for 30 min, stained with naphthalen blue black and washed once with tap water. Plaques were counted and the titer for 50% plaque reduction was calculated referred to VACV-negative serum.

### NT-PCR assay

To optimize the NT-PCR assay, three VACV LE concentrations (4.4 × 10^2^, 2.2 × 10^2 ^and 4.4 × 10^1 ^pfu/well), a neutralization time between 1 h, 2 h and 3 h, total incubation time for virus entry and initial replication of 24 h, 7 h or 5 h and three different viral target sequences for OPV-specific real-time PCR were investigated. All other preceding steps were performed according to the standard PRNT. After incubation, medium was aspirated and cells were washed with PBS, trypsinized, washed twice with PBS and centrifuged (5 min, 250 × g). Cell pellet was immediately resuspended in 700 μL RLT buffer and RNA was extracted using the RNeasy Mini Kit (Qiagen, Hilden, Germany) according to the manufacturer's protocol. Residual DNA was eliminated by DNase treatment (Turbo DNase Kit; Ambion, Hamburg, Germany). cDNA was synthesized in a total volume of 20 μL containing 500 ng Oligo dT_(18) _primer, 500 ng calf thymus DNA and 10 μL RNA. No RNase inhibitors were added as all RNA samples were processed immediately after extraction. After 5 min at 65°C a mix consisting of 4 μL 5 × buffer, 2 μL 0.1 M DTT, 0.4 μL 25 mM dNTP and 200 U Superscript II reverse transcriptase (Invitrogen, Karlsruhe, Germany) was added.

Samples were incubated at 37°C for 50 min and at 70°C for 15 min. For each RNA sample a reverse transcription (RT) control reaction was performed replacing the reverse transcriptase water to verify absence of contaminating DNA. Quality of cDNAs was tested using the c-myc real-time PCR assay which simultaneously served as control of RNA extraction and cDNA synthesis. Only cDNA with C_T _values ≥ 10^4 ^c-myc copies per reaction were further analyzed. Four real-time PCR assays targeting different viral genes (table 4) were established and tested to quantify VACV gene expression as indicator for active virus replication. The respective amplicons of all real-time PCRs were cloned into pCR2.1 vector, according to the manufacturer's instructions (TOPO TA Cloning Kit, Invitrogen, Karlsruhe, Germany). Plasmids were purified, copy numbers calculated and tenfold serially diluted in a background of 1 ng/μL λ DNA (Fermentas, St. Leon-Rot, Germany). Samples were measured in duplicate and mean C_T _values were used to calculate copy numbers using 7900 and 7500 real-time PCR instruments (Applied Biosystems, Darmstadt, Germany). The 25 μL reaction volume included 2 μL cDNA or plasmid DNA, 2.5 μL 10 × buffer, 2.0 μL MgCl_2 _(50 mM), 1 μL dNTP (25 mM each), 0.75 μL of forward and reverse primers (10 μM), 0.25 μL probe (10 μM), 0.25 μL ROX (100 mM), 1 U Platinum Taq polymerase (Invitrogen, Karlsruhe, Germany) and 15.3 μL water. Cycling conditions were 2 min at 50°C, 10 min at 95°C, followed by 45 cycles of 15 sec at 95°C and 30 sec at 60°C (62°C, OPV_12/13_).

**Table 4:** Details of oligo nucleotides used in PCR reactions

Oligo name	Oligonucleotide sequence 5'→3'	S/A	Ta (°C)	Tm (°C)
**c-myc**			**60.0**	
c-myc F	GCCAGAGGAGGAACGAGCT	S		59.4
c-myc R	GGGCCTTTTCATTGTTTTCCA	A		54.2
c-myc TM	F-TGCCCTGCGTGACCAGATCC-T	S		65.9
**OPV_12/13_/MPXV** **82 protein**			**62.0**	
OPV F	GCCAATTGTCTTTCTCTTTTACTGA	S		56.2
OPV R	GAAAACATTTAAGGATGAATCCATCT	A		55.4
OPV TMGB	F-CCTTCTATAGATCTGAGAAT NQF MGB	S		65.5
**rpo18/polymerase^#^**			**60.0**	
rpo18 F	CTGTAGTTATAAACGTTCCGTGTG	S		50.7
rpo 18 R	TTATCATACGCATTACCATTTCGA	A		47.0
rpo18 TM	F-ATCGCTAAATGATACAGTACCCGAAXTCTCTACT-PH			
**D8L/IMV membrane protein**			**60.0**	
D8L F	GGATGTTCTATATACGGGGATGAGTAG	S		57.9
D8L R	AAAGTTAATAAGGTAGATGACACGTTCT	A		58.5
D8L TM	F-TTCTCATCATCAGAATAAA-NFQ MGB	S		65.1

**Y **= C or T, **B **= C or G or T, **W **= A or T, **F **= FAM label, **T **= TAMRA label, **TM **= hydrolysis probe, **TMGB **= hydrolysis probe coupled to an MGB moiety; **MGB **= Minor Groove Binder, **NFQ **= Non-fluorescent quencher, **Tm **= Melting temperature (calculated by nearest neighbor method), **Ta **= Annealing temperature, **A **= antisense orientation, **S **= sense orientation, # [7].

Statistical analyses were done using GraphPad's Prism software (Version 5.0a) and a repeated measures ANOVA test and "Tukey's Multiple Comparison Test" was used for statistical analysis and p-value ≤ 0.05 was regarded as significant.

## List of Abbreviations

HVIG: human Vaccina virus immunoglobulin, OPV: orthopox virus, PRNT: plaque-reduction neutralization test, VACV: Vaccinia virus, VACV LE: Vaccinia virus Lister Elstree

## Competing interests

The authors declare that they have no competing interests.

## Authors' contributions

MK made substantial contributions to the study design, participated in data acquisition, analysis and interpretation and drafted and revised the manuscript critically. AD was mainly responsible for data acquisition and analysis and for interpretation of data. GFL was involved in data analysis and in discussing the manuscript. PWD participated in data analysis and interpretation. HE made substantial contributions to conception and design of the experiment and in revising the manuscript. All authors read and approved the final manuscript.
